# Suggestive causal associations between 17 autoimmune diseases and pancreatitis: A two-sample Mendelian randomization study

**DOI:** 10.1097/MD.0000000000050766

**Published:** 2026-09-18

**Authors:** Dehua Huang, Yun Li, Zhenyu Li, Chongfa Chen, Huijuan Wang, Hanxiao You, Jinfan Zhang, Shangnan Dai, Guangfu Wang, Chunhui Lu, Chenchen Li, Feihu Sun, Lingdi Yin, Yi Miao

**Affiliations:** a Pancreas Center, The Affiliated BenQ Hospital of Nanjing Medical University, Nanjing, Jiangsu, China; c Pancreas Center, The First Affiliated Hospital of Nanjing Medical University, Nanjing, Jiangsu, China; b Pancreas Institute of Nanjing Medical University, Nanjing, Jiangsu, China; d Department of Rheumatology, The First Affiliated Hospital of Nanjing Medical University, Nanjing, China.

**Keywords:** autoimmune diseases, Mendelian randomization, pancreatitis, primary sclerosing cholangitis, type 1 diabetes, ulcerative colitis

## Abstract

The relationship between different autoimmune diseases (ADs) and pancreatitis has been reported in epidemiological studies. This study systematically investigated the causal effects of ADs factors on pancreatitis using Mendelian randomization (MR). We selected 17 common ADs as exposures from the largest genome-wide association study dataset in Europe. The outcome variables included acute pancreatitis (AP), chronic pancreatitis (CP), alcohol-induced acute pancreatitis, and alcohol-induced chronic pancreatitis. A variety of analytical methods were used to comprehensively evaluate the causal relationship between ADs and various types of pancreatitis, with sensitivity analyses, including Cochran’s *Q* test and MR-Egger intercept test performed to verify the reliability of the results. Finally, a reverse MR analysis was performed to evaluate the possibility of reverse causation. Genetically predicted type 1 diabetes (OR = 1.032, 95% CI = 1.008–1.056, P = .0086) and ulcerative colitis (OR = 1.065, 95% CI = 1.012–1.121, *P* = .0151) were associated with an increased risk of AP. primary sclerosing cholangitis (OR = 1.080, 95% CI = 1.013–1.153, P = .0194) increased the risk of CP. Reverse MR analysis suggested a potential protective causal association between alcohol-induced acute pancreatitis and hashimoto’s thyroiditis (OR = 0.962, 95% CI = 0.936–0.989, *P* = .0056), although this finding should be regarded as suggestive because it did not remain statistically significant after Bonferroni correction. Sensitivity analysis did not detect horizontal pleiotropy or heterogeneity. Genetic evidence supports a causal link between type 1 diabetes, ulcerative colitis, and AP, as well as between primary sclerosing cholangitis and CP. This analysis provides new insights into ADs as risk factors for pancreatitis, but further experimental research is needed to explore the potential causal relationships between these factors.

## 1. Introduction

Pancreatitis, caused by the auto-digestive action of pancreatic proteases, is a complex and potentially life-threatening gastrointestinal emergency, particularly in developed countries.^[1]^ acute pancreatitis (AP) occurs at an incidence of approximately 34 cases per 100,000 people in high-income countries,^[2]^ and up to 20% of cases progress to moderate or severe forms with substantial morbidity and mortality. Approximately 3% to 35% of patients develop chronic pancreatitis (CP) within several years,^[3–5]^ which can result in persistent abdominal pain, pancreatic insufficiency, diabetes, and pancreatic cancer.^[6]^ Alcohol consumption is a major risk factor and is more commonly associated with pancreatitis in Western countries and Japan than in other Asian regions,^[7]^ leading to 2 subtypes: alcohol-induced acute pancreatitis (AAP) and alcohol-induced chronic pancreatitis (ACP).

In addition to well-established risk factors such as gallstones, alcohol, and hyperlipidemia,^[7,8]^ autoimmune diseases (ADs) have drawn increasing attention as potential contributors to pancreatitis. Epidemiological studies have suggested an association between pancreatitis and various ADs, including rheumatoid arthritis (RA), ulcerative colitis (UC), celiac disease (CeD), Hashimoto’s thyroiditis, and primary biliary cirrhosis.^[9–16]^ However, these observational findings are vulnerable to residual confounding and reverse causality, and the causal nature of these associations remains unclear.

Mendelian randomization (MR) leverages genetic variants as instrumental variables to strengthen causal inference.^[17–21]^ While previous MR analyses have explored selected immune-mediated diseases, such as inflammatory bowel disease in relation to pancreatitis,^[22]^ a comprehensive evaluation across multiple ADs and distinct forms of pancreatitis is lacking. Given the possible heterogeneity in pathophysiology, this study investigated the genetic causal relationships between 17 common ADs and 4 clinically important pancreatitis subtypes: AP, CP, AAP, and ACP, representing both acute and chronic, alcohol-related and non-alcohol-related forms. This approach may help clarify shared biological pathways and inform preventive strategies.

## 2. Methods study design

**Figure 1.**
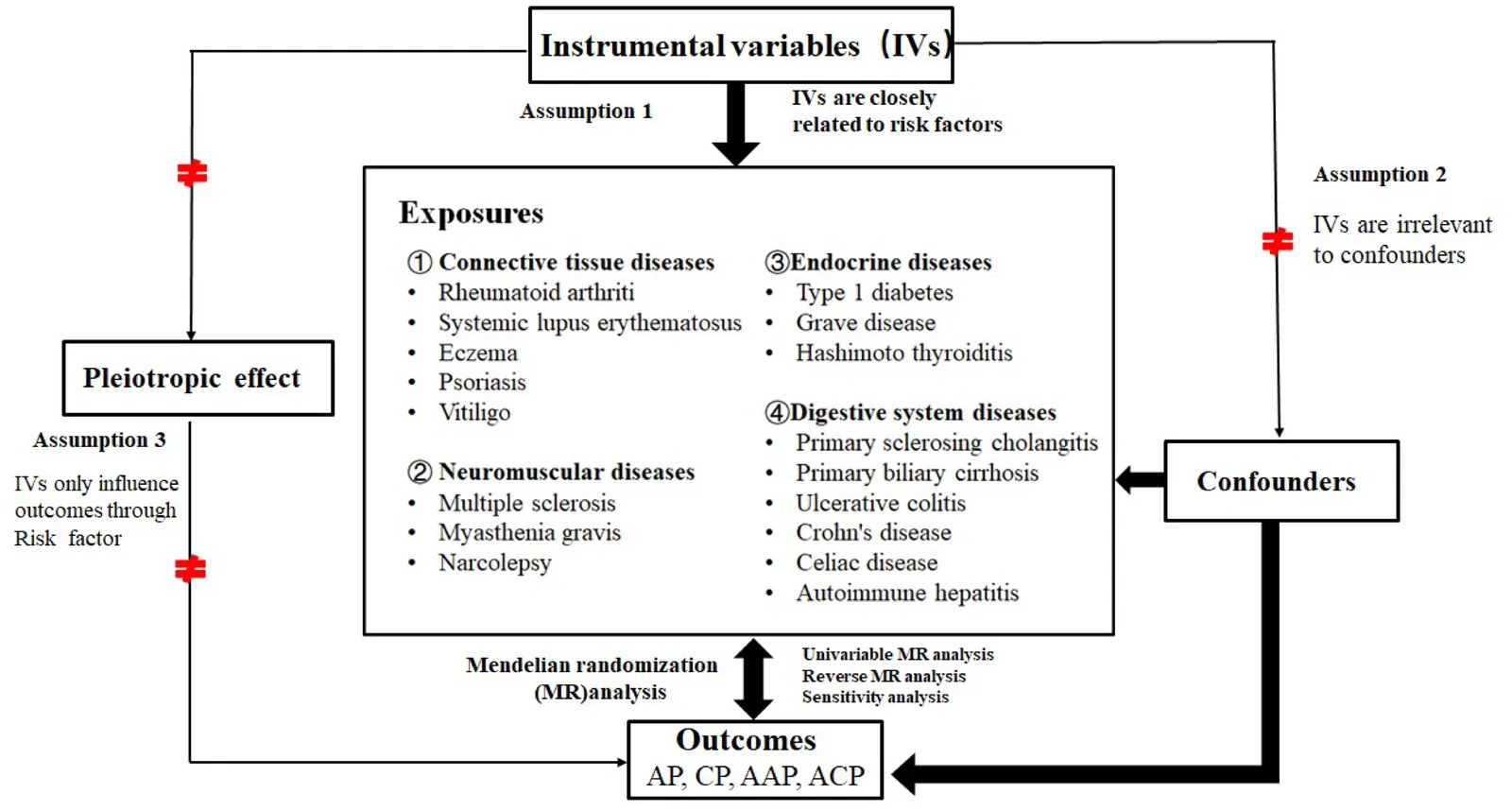
Flowchart of the MR study between pancreatitis and 17 ADs. AAP = alcohol-induced acute pancreatitis, ADs = autoimmune diseases, ACP = alcohol-induced chronic pancreatitis, CP = chronic pancreatitis, MR = Mendelian randomization, AP = acute pancreatitis.

MR was used to study the causal relationship between ADs and the different types of pancreatitis. Single nucleotide polymorphisms (SNPs) associated with these risk factors were used as instrumental variables (IVs). The following 3 assumptions served as the foundation for the MR study: the SNPs are closely related to the risk factors, the SNPs are irrelevant to various confounders, and the SNPs only influence the outcomes through the risk factors (Fig. 1). Since the data used were based on published research and public databases (genome-wide association study (GWAS), European genome-wide association studies, and Finngen datasets open to IEU), there was no need for additional ethical approval from the Institutional Review Committee.

### 2.1. Study cohorts and GWAS

Seventeen ADs closely related to pancreatitis were selected for use in this analysis (Literature Retrieval Process ADs Related to Pancreatitis in Table 1). In addition, datasets for each AD were obtained from the GWAS and Finngen datasets open to IEU as exposures, including RA, systemic lupus erythematosus (SLE), Eczema, Psoriasis, Vitiligo, multiple sclerosis (MS), myasthenia gravis (MG), narcolepsy, type 1 diabetes (T1D), graves’ disease (GD), hashimoto’s thyroiditis (HT), primary sclerosing cholangitis (PSC), primary biliary cirrhosis (PBC), UC, crohn’s disease (CD), CeD, and autoimmune hepatitis (AH). The diseases were classified into 4 categories: connective tissue diseases, neuromuscular diseases, endocrine diseases, and digestive system diseases. The outcomes were 4 different types of pancreatitis: AP, CP, AAP, and ACP. The latest and largest publicly available GWAS summary data were used; all case and control groups were of European origin, and there was no significant overlap between GWAS populations (Table 2). Preliminary studies have provided detailed information on registration procedures and diagnostic criteria.

**Table 1:** Literature retrieval process autoimmune diseases related to pancreatitis.

Database	Retrieval strategies	Merge
PubMed	#1“Pancreatitis”[Mesh] OR “Acute pancreatitis” OR “Chronic pancreatitis” OR “Alcohol induced acute pancreatitis” OR “Alcohol induced chronic pancreatitis” #2 “Autoimmune Diseases” [Title/Abstract] OR “Autoimmunity “[Mesh] OR “Rheumatoid Arthritis”[Mesh] OR “Systemic Lupus Erythematosus”[Mesh]]OR “Multiple Sclerosis”[Mesh] OR “Type 1 Diabetes”	#1 AND #2
Embase	#1 “pancreatitis*”:ab,ti OR “acute pancreatitis *”:ab,ti OR “Chronic pancreatitis”:ab,ti OR “Alcohol induced acute pancreatitis”:ab,ti OR “Alcohol induced chronic pancreatitis”/ exp #2 “autoimmune disease”:ab,ti OR “autoimmunity”/exp.OR “rheumatoid arthritis”:ab,ti OR “systemic lupus erythematosus”:ab,ti OR “multiple sclerosis,”:ab,ti OR “type 1 diabetes”:ab,ti	#1 AND #2
Scopus	#1 TITLE-ABS-KEY (“Pancreatitis*”) OR TITLE-ABS-KEY (“Acute pancreatitis *”)OR TITLE-ABS-KEY (“Alcohol induced acute pancreatitis*”) OR TITLE-ABS-KEY (“Alcohol induced chronic pancreatitis*”) #2 TITLE-ABS-KEY (“autoimmune diseases”) OR TITLE-ABS-KEY (“autoimmunity “) OR TITLE-ABS-KEY(“rheumatoid arthritis”) OR TITLE-ABS-KEY(“autoimmune hepatitis”) OR TITLE-ABS-KEY(“systemic lupus erythematosus”) OR TITLE-ABS-KEY(“multiple sclerosis”) OR TITLE-ABS-KEY (“systemic lupus erythematosus”) OR TITLE-ABS-KEY (“type 1 diabetes”)	#1 AND #2

**Table 2:** Characteristics of the ADs and pancreatitis GWAS cohorts.

Disease	Study	Journal	Cases	Controls	Sample size	GWAS ID
RA	Sakaue S et al.2021	Nat Genet	8255	409,001	417,256	ebi-a-GCST90018910
SLE	Sakaue S et al.2021	Nat Genet	647	482,264	482,911	ebi-a-GCST90018917
Eczema	Loh PR et al.2018	Nat Genet	NA	NA	461,199	ebi-a-GCST90029017
Psoriasis	Stuart PE al.2022	HGG Adv	15,967	28,194	44,161	ebi-a-GCST90019016
Vitiligo	NA	NA	131	207,482	NA	finn-b-L12_VITILIGO
MS	Andlauer TF et al.2016	Sci Adv	4888	10,395	15,283	ebi-a-GCST003566
MG	Sakaue S et al.2021	Nat Genet	197	354,945	355,142	ebi-a-GCST90018876
Narcolepsy	Faraco J et al.2013	PLoS Genet	1886	10,421	12,307	ebi-a-GCST005522
T1D	Chiou J et al.2021	Nature	18,942	501,638	520,580	ebi-a-GCST90014023
GD	Sakaue S et al.2021	Nat Genet	1678	456,942	458,620	ebi-a-GCST90018847
HT	Sakaue S et al.2021	Nat Genet	15,654	379,986	395,640	ebi-a-GCST90018855
PSC	Sun-Gou Ji et al.2017	Nat Genet	2871	12,019	14,890	ieu-a-1112
PBC	Liu JZ et al.2012	Nat Genet	2861	8514	11,375	ebi-a-GCST005581
UC	Saori Sakaue et al.2021	Nat Genet	5371	412,561	417,932	ebi-a-GCST90018933
CD	Neale et al.2017	NA	732	336,467	337,199	ukb-a-552
CeD	Trynka et al.2017	Nat Genet	12,041	12,228	24,269	ieu-a-1058
AH	Sakaue S et al.2021	Nat Genet	821	484,413	485,234	ebi-a-GCST90018785
AP	Sakaue S et al.2021	Nat Genet	3798	476,104	479,902	ebi-a-GCST90018789
CP	Sakaue S et al.2021	Nat Genet	1424	477,528	477,528	ebi-a-GCST90018821
AAP	NA	NA	457	218,335	NA	finn-b-ALCOPANCACU
ACP	NA	NA	977	217,815	NA	finn-b-ALCOPANCCHRON

AAP = alcohol-induced acute pancreatitis, ACP = alcohol-induced chronic pancreatitis, AH = autoimmune hepatitis, AP = acute pancreatitis, CD = crohn’s disease, CeD = celiac disease, CP = chronic pancreatitis, GD = graves’ disease, HT = hashimoto’s thyroiditis, MG = myasthenia gravis, MS = multiple sclerosis, PBC = primary biliary cholangitis, PSC = primary sclerosing cholangitis, RA = rheumatoid arthritis, SLE = systemic lupus erythematosus, T1D = type 1 diabetes, UC = ulcerative colitis.

### 2.2. Instrumental variables selection

SNPs were selected for each AD, and default settings in the “TwoSampleMR” R package were used. Genome-wide significant SNPs were selected based on a *P*-value < 5 × 10^−6^, and standard clumping parameters were used to select independent SNPs (clumping window of 10,000 kb, LD *r*^2^ cutoff 0.001). The intensity of the instrumental variables (IVs) was estimated by calculating the proportion of variance in the disease explained by SNPs *(R^2^*), and the F-statistic was used to satisfy the first MR hypothesis. *F* statistics (*F* = beta^2^/se^2^) were calculated for each SNP, and a general F statistic was calculated for all SNPs for the corresponding exposure.^[23]^*F* > 10 was considered to indicate sufficient strength. Reverse MR imaging was performed using the same approach.

### 2.3. Mendelian randomization analysis

Two-sample MR analyses were conducted using random-effects inverse-variance weighted (IVW),^[23]^ weighted median, and MR-Egger. The traditional IVW analysis method may be affected by invalid instrumental bias or multivariate validity; therefore, the present study conducted sensitivity analysis to test IVW. Validity and robustness of results. To minimize the bias caused by horizontal pleiotropy (affecting the results through causal pathways rather than exposure), this study used pleiotropic residual sums and outliers (MR-PRESSO) to test for extensive horizontal pleiotropy. Two other established MR methods, including weighted median and MR-Egger regression methods, were used for the association between ADs and pancreatitis. Further tests were conducted; finally, to assess the robustness of the results, further heterogeneity tests, including the MR-Egger intercept test, sensitivity analysis, and modified Cochrane’s *Q* statistic, were performed for statistically significant results.^[23]^ Finally, a leave-one-out analysis was performed to screen for any individual SNPs that were disproportionately accountable for the outcome of each MR. As the outcome was a dichotomous variable, the effect estimates were converted to odds ratios (OR) to visualize the relationship between ADs and pancreatitis ^[23]^.pancreatitis.^[23]^ Statistical significance was set at *P* < .05.

Multiple hypothesis test corrections were performed using the Bonferroni correction. A Bonferroni-corrected significance level of *P* < 2.94 × 10^−3^ (0.05/17) was used, and *P* values ranging from 2.78 × 10^−3^ to .05 were classified as suggestive causal associations. All MR analyses were implemented using the 2 Sample MR package version 0.5.8 in *R* (version 4.3.1).

## 3. Results

### 3.1. Causal effects of 17 autoimmune diseases on acute pancreatitis

**Figure 2.**
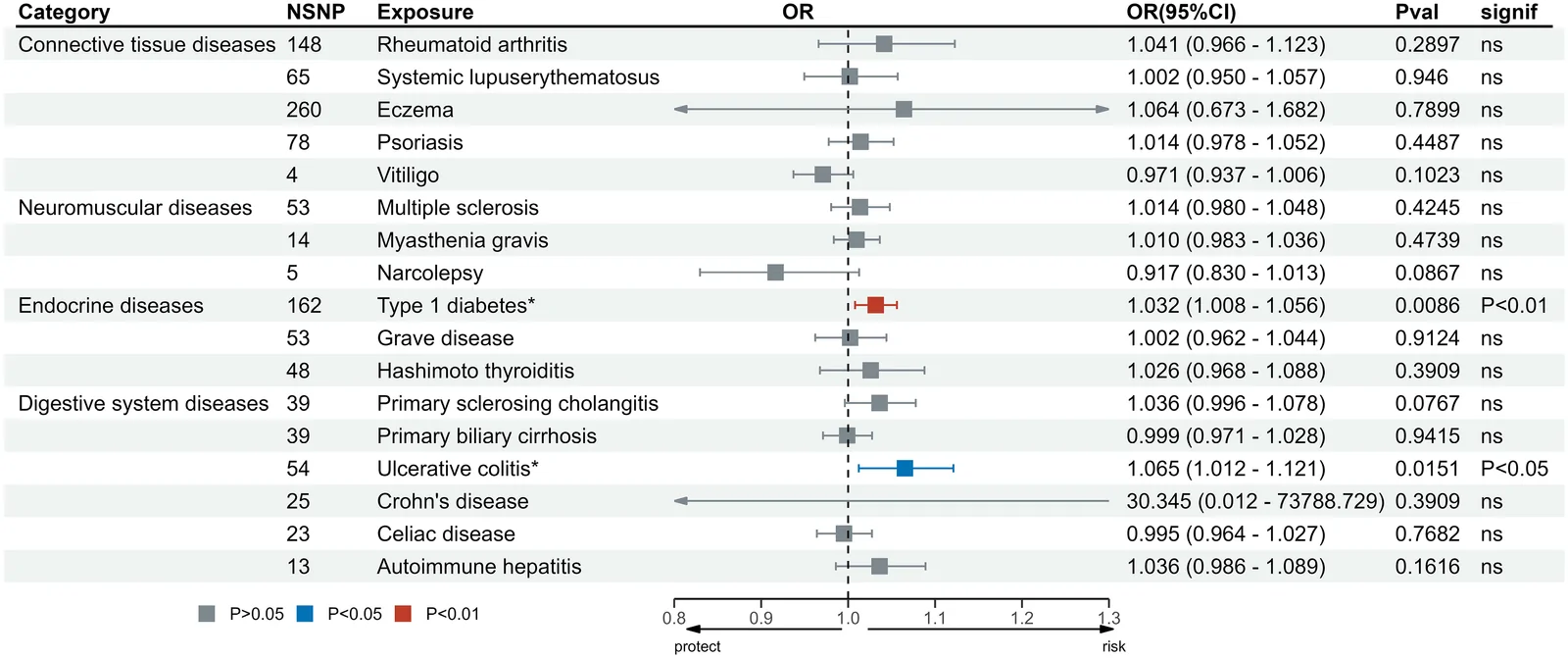
Forest plot to visualize the causal effect of 17 ADs on AP using the inverse variance-weighted method. ADs = autoimmune diseases, AP = acute pancreatitis.

The IVW analysis revealed that only T1D and UC were causally associated with AP (T1D: OR = 1.032, 95CI% = 1.008–1.056, *P* = .0086; UC: OR = 1.065, 95CI% = 1.012–1.121, *P* = .0151). Other findings were that all 11 ADs increased the risk of AP (RA: OR = 1.041, *P* = .2897; SLE: OR = 1.002, *P* = .946; Eczema: OR = 1.064, *P* = .7899; Psoriasis: OR = 1.014, *P* = .4487; MS: OR = 1.014, *P* = .4245; MG: OR = 1.010, *P* = .4739; GD: OR = 1.002, *P* = .9124; HT: OR = 1.026, *P* = .3909; PSC: OR = 1.036, *P* = .0767; CD: OR = 30.345, *P* = .3909; AH: OR = 1.036, *P* = .1616), but there was no causality (Fig. 2).The weighted median, MR-Egger test, pleiotropy, heterogeneity, and leave-one-out results are presented in Supplementary Figure 1, Supplemental Digital Content 1 and Supplementary Table 1, Supplemental Digital Content 3.

### 3.2. Causal effects of 17 autoimmune diseases on chronic pancreatitis

**Figure 3.**
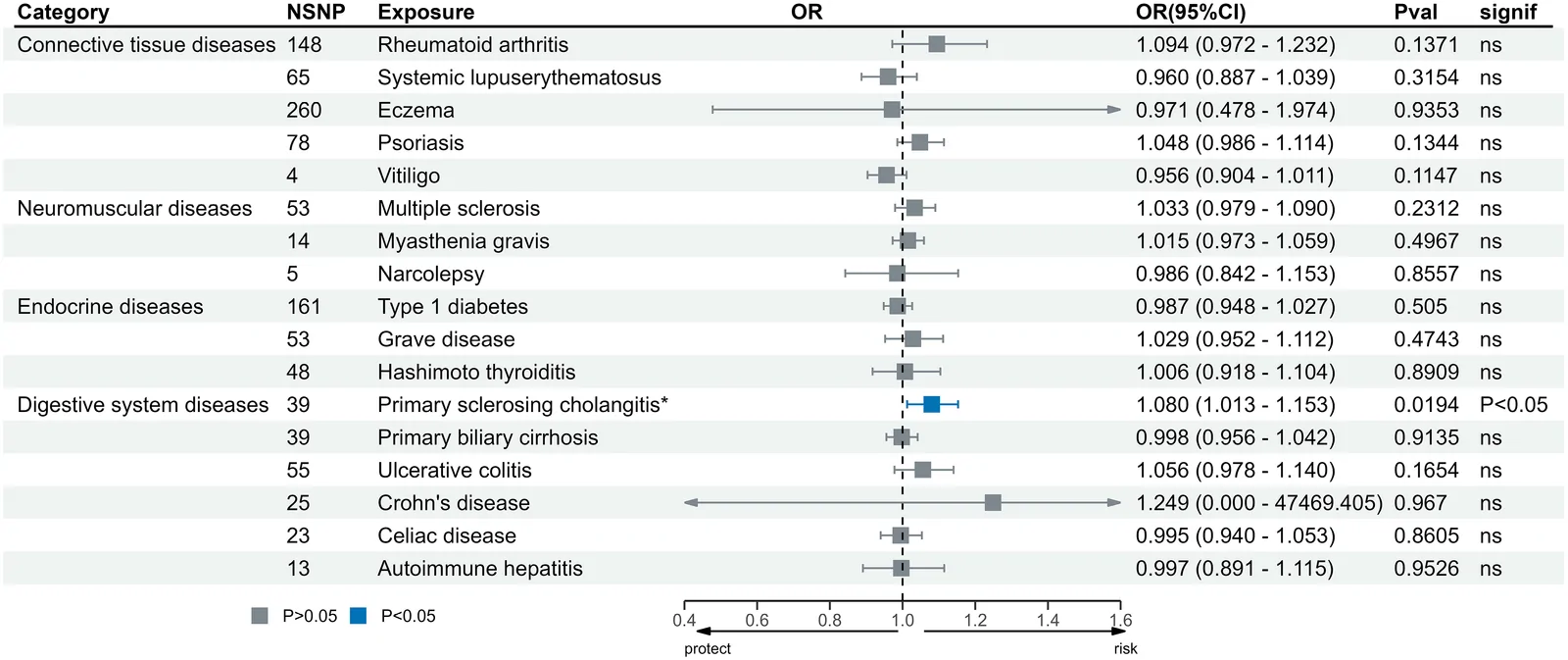
Forest plot to visualize the causal effect of 17 ADs on CP using the inverse variance-weighted method. ADs = autoimmune diseases, AP = acute pancreatitis.

The IVW analysis showed that only PSC was causally associated with CP (PSC: OR = 1.080, 95% CI = 1.013–1.153, *P* = .0194). Other results showed 7 ADs increased the risk of CP (RA: OR = 1.094, *P* = .1371; Psoriasis: OR = 1.048, *P* = .134; MS: OR = 1.033, *P* = .2312; MG: OR = 1.015, *P* = .4967; GD: OR = 1.029, *P* = .4743; UC: OR = 1.056, *P* = .1654; CD: OR = 1.249, *P* = .967), but there was no causal relationship (Figure 3).The weighted median, MR-Egger test, pleiotropy, heterogeneity, and leave-one-out results are presented in Supplementary Figure 1, Supplemental Digital Content 1 and Supplementary Table 2, Supplemental Digital Content 4.

### 3.3. Causal effects of 17 autoimmune diseases on alcohol-induced pancreatitis

**Figure 4.**
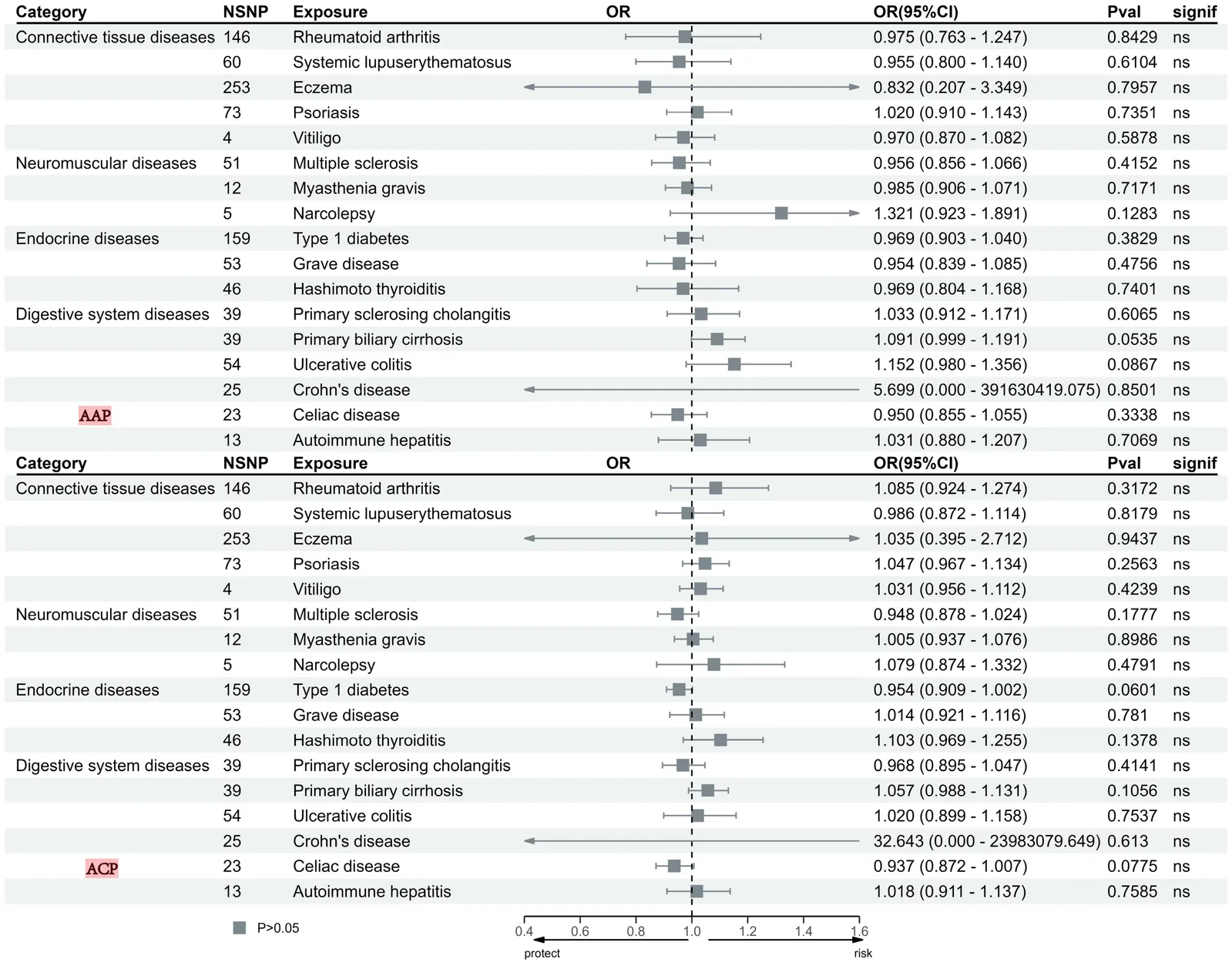
Forest plot to visualize the causal effect of 17 ADs on alcohol-induced pancreatitis using the inverse variance-weighted method. ADs = autoimmune diseases, AP = acute pancreatitis.

The IVW analysis showed no causal relationship between ADs and alcohol-induced pancreatitis. Other results were that 7 ADs increased the risk of AAP (Psoriasis: OR = 1.020, *P* = .7351; Narcolepsy: OR = 1.321, *P* = .1283; PSC: OR = 1.033, *P* = .6065; PBC: OR = 1.091, *P* = .0535; UC: OR = 1.152, *P* = .0867; CD: OR = 5.699, *P* = .8850; AH: OR = 1.031, *P* = .7069), and the presence of 12 ADs was associated with an increased risk of ACP (RA: OR = 1.085, *P* = .3171; Eczema: OR = 1.035, *P* = .9437; Psoriasis: OR = 1.047, *P* = .2563; Vitiligo: OR = 1.031, *P* = .4239; MG: OR = 1.005, *P* = .8986; Narcolepsy: OR = 1.079, *P* = .4791; GD: OR = 1.014, *P* = .781; HT: OR = 1.103, *P* = .1378; PBC: OR = 1.057, *P* = .1056; UC: OR = 1.020, *P* = .7537; CD: OR = 32.643, *P* = .613; AH: OR = 1.018, *P* = .7585), but there was no causal relationship (Fig. 4) The weighted median, MR-Egger test, pleiotropy, heterogeneity, and leave-one-out results are presented in Supplementary Figure 1, Supplemental Digital Content 1 and Supplementary Tables 3, Supplemental Digital Content 5 and 4, Supplemental Digital Content 6.

### 3.4. Reverse MR analysis: causal effects of 17 autoimmune diseases on pancreatitis

**Figure 5.**
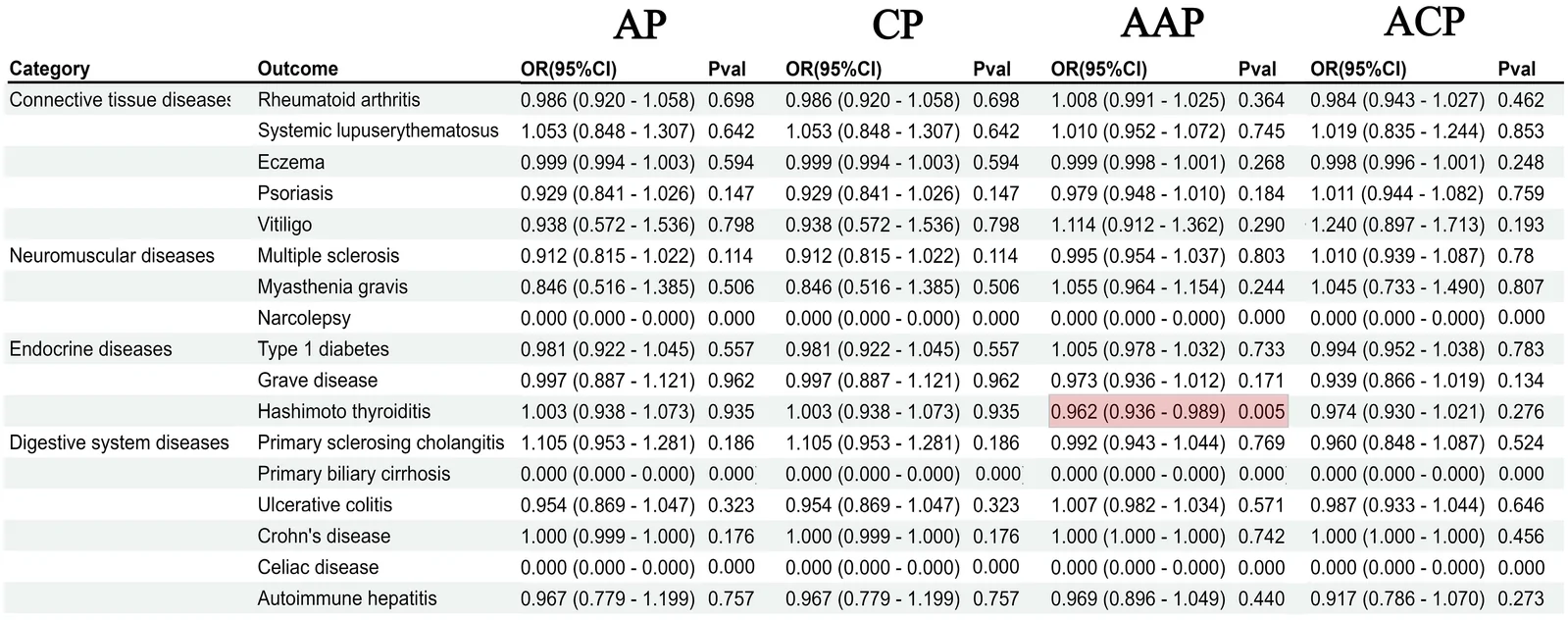
Summary data to visualize the causal effect of 4 Pancreatitis on 17 ADs using the inverse variance-weighted method. AAP = alcohol-induced acute pancreatitis, ACP = alcohol-induced chronic pancreatitis, ADs = autoimmune diseases, AP = acute pancreatitis, CP = chronic pancreatitis.

Reverse MR analysis showed that only AAP was potentially associated with a lower risk of HT (OR = 0.962, 95% CI = 0.936–0.989, *P* = .0056). AP, CP, AAP, and ACP were not causally associated with 17 ADs (Fig. 5)The IVW results are shown in Supplementary Figure 2, Supplemental Digital Content 2 and Supplementary Table 5, Supplemental Digital Content 7.

## 4. Discussion

ADs are caused by the immune system’s inability to recognize foreign invaders and self-antigens,^[24]^ thus, inflammatory mediators (inflammatory factors and inflammasomes) play an important role in the pathogenesis of ADs. Innate immune cells (macrophages and granulocytes) proliferate abnormally, secrete large amounts of inflammatory factors, and stimulate the abnormal infiltration of T and B cells, causing progressive inflammation and tissue damage.^[25,26]^ Pancreatitis is caused by the self-digestion of pancreatic tissue by trypsin. Under normal circumstances, trypsinogen in the pancreatic juice is inactive. When influenced by certain factors, such as gallstones, alcoholism, high triglycerides, infection, immune diseases, or drug toxicity, inflammation occurs, causing trypsin to be activated, followed by other enzyme reactions such as elastase and phospholipase A, which cause self-digestion of the pancreas and promote its necrosis and dissolution. Pancreatitis has obvious complications and a high mortality rate, with a mortality rate of approximately 2% to 5%.^[27]^ The occurrence and progression of pancreatitis are multifaceted, resulting from the interactions between genetic and environmental factors. Epidemiological studies have reported that various ADs increase the risk of pancreatitis; however, the causal impact of genetics on ADs and pancreatitis is still unclear.

### 4.1. Acute pancreatitis (AP)

Our analysis demonstrated that genetically predicted type 1 diabetes mellitus (T1DM) and UC) were associated with an increased risk of AP. This is in line with previous epidemiological studies reporting a higher incidence of AP in patients with ADs such as RA, UC, and CeD. For connective tissue diseases (RA, SLE, psoriasis, eczema, and vitiligo),^[28]^ however, we did not observe genetic causality with pancreatitis. Notably, 2 large population-based cohort studies from Taiwan showed that RA was associated with an increased AP risk (HR = 1.62; 95% CI: 1.43–1.83),^[9]^ and psoriasis with an increased CP risk (HR = 1.81; 95% CI: 1.53–2.15), but these risks were reduced with corticosteroids, nonsteroidal anti-inflammatory drugs, or methotrexate treatment.^[29]^ Conversely, drug withdrawal and the use of certain disease-modifying antirheumatic drugs have also been linked to increased AP risk.^[30,31]^ These contradictory findings suggest that pharmacological factors, rather than genetic predisposition, may play a more important role in the development of pancreatitis in connective tissue diseases.

With regard to T1DM, biological plausibility is supported by shared autoimmune pathways.^[32]^ T1DM frequently coexists with CeD owing to common HLA haplotypes, while non-hla genetic and environmental factors (viral infections, gut microbiota, and gluten exposure) may also contribute. Pancreatic *β*-cells aberrantly express tissue transglutaminase under stress, potentially linking T1DM autoimmunity to exocrine pancreatic injury. Although autoimmune pancreatitis (AIP) is exceedingly rare in children and is not considered a relevant comorbidity of CeD, immune-mediated overlap may exist and warrant further investigation.^[33]^ Our findings confirm that T1DM increases the risk of AP, supporting the hypothesis that immune dysregulation in diabetes may extend to the pancreatic exocrine tissues.

Observational studies have suggested a 2 to 3-fold higher risk of pancreatitis for CeD, potentially due to malnutrition-related acinar atrophy, altered intestinal hormone secretion, and shared Th1-driven immune responses. However, our MR results do not support a causal link. Importantly, a recent pediatric review concluded that pancreatitis, including autoimmune forms, is not a relevant comorbidity in children with CeD, highlighting possible age-related differences.

### 4.2. Chronic pancreatitis (CP)

We identified a causal genetic association between PSC and CP. This is biologically plausible, as PSC shares immunopathological features with AIP including elevated serum IgG4 levels, which have been reported in 9% to 36% of patients with PSC, and lymphoplasmacytic infiltration of hepatobiliary tissues.^[34]^ High IgG4 levels have also been observed in the liver, gallbladder, and extrahepatic bile ducts, which are commonly affected by AIP. Whether PSC and AIP develop independently or represent variations within the same IgG4-related disease spectrum remains uncertain, but their immunological overlap provides mechanistic support for our findings.^[35]^

In contrast, although psoriasis has been associated with CP in observational studies, our MR analysis did not confirm a genetic relationship, suggesting that environmental or therapeutic factors may account for the observed risk. Similarly, no causal associations were detected between CP and other autoimmune conditions such as HT or primary biliary cholangitis (PBC).^[36]^ Previous studies have suggested immunological overlaps between primary biliary cholangitis and AIP including HLA-DQB1 susceptibility, dysregulation of cytotoxic T-lymphocyte antigen-4, predominant T-cell–mediated mechanisms, and B-cell infiltration.^[16,37]^ Nevertheless, our MR analysis did not reveal a genetic relationship between primary biliary cholangitis and pancreatitis, and comorbidity has only been described in isolated case reports.

### 4.3. Alcohol-related pancreatitis (AAP and ACP)

Alcohol is a well-established risk factor for pancreatitis; however, its interaction with ADs remains poorly understood. In the present reverse MR analysis, we observed a suggestive protective causal association between AAP and HT. Specifically, genetically predicted AAP was associated with a lower risk of HT. However, this association did not remain statistically significant after Bonferroni correction and therefore should be interpreted with caution.

The biological mechanisms underlying this observation remain unclear. Given the exploratory nature of this finding, it is premature to infer a direct protective effect of AAP on thyroid autoimmunity. Instead, the observed association may reflect shared genetic backgrounds, immune regulatory pathways, or other unmeasured factors that warrant further investigation.^[36]^ Future experimental and epidemiological studies are needed to validate this finding and clarify the underlying mechanisms. No significant associations were observed between other ADs and alcohol-related pancreatitis, underscoring the need for further validation in larger and more diverse populations.

### 4.4. Reverse Mendelian Randomization findings

In addition to the forward MR results, reverse analysis indicated that AAP may increase the risk of HT, suggesting a bidirectional relationship between alcohol-related pancreatic inflammation and thyroid autoimmunity. However, no other reverse causal links have been identified between pancreatitis subtypes and ADs.

### 4.5. Strengths and limitations

This is the first bidirectional MR study to systematically evaluate the causal relationship between 17 ADs and 4 pancreatitis subtypes. The bidirectional approach strengthens causal inference and reduces residual confounding compared with observational studies. Nevertheless, this study has several limitations that must be acknowledged. First, our datasets were derived exclusively from European populations, which may limit their generalizability to other ethnic groups. Second, sex-specific analyses could not be performed, although many ADs are more prevalent in women. Finally, only 4 pancreatitis subtypes (AP, CP, AAP, and ACP) were analyzed because of the absence of large-scale GWAS data for other clinically relevant forms such as AIP and acute recurrent pancreatitis (ARP). Future studies that include more diverse populations, sex-stratified analyses, and expanded GWAS resources are warranted to validate and extend these findings.

## 5. Conclusion

This MR study suggests potential genetic associations between certain ADs and pancreatitis in European populations. In particular, T1DM and UC may have causal links with AP, while PSC appears related to CP. No strong genetic evidence was found for other ADs. These results warrant cautious interpretation and further confirmation in other populations and pancreatitis subtypes.

## Acknowledgments

The authors acknowledge the UK Biobank, FinnGen Biobank, Sakaue S et al., Loh PR et al., Stuart PE et al., Faraco J et al., Chiou J et al., Andlauer TF et al., and others, for their publicly available summary data.

## Author contributions

**Conceptualization:** Dehua Huang, Zhenyu Li, Yi Miao.

**Data curation:** Dehua Huang, Zhenyu Li, Chunhui Lu, Chenchen Li.

**Investigation:** Zhenyu Li, Huijuan Wang.

**Methodology:** Zhenyu Li, Shangnan Dai, Lingdi Yin.

**Formal analysis:** Chongfa Chen, Guangfu Wang, Feihu Sun.

**Software:** Hanxiao You.

**Supervision:** Jinfan Zhang.

**Funding acquisition:** Yi Miao.

**Writing – original draft:** Dehua Huang, Yun Li, Zhenyu Li, Yi Miao.














