# Supplementary material for: Suggestive causal associations between 17 autoimmune diseases and pancreatitis: A two-sample Mendelian randomization study
Source: Medicine (Baltimore). 2026 Sep 18;105(38):e50766. doi: 10.1097/MD.0000000000050766 (PMC13593168; doi:10.1097/MD.0000000000050766)
Supplement: Supplementary file 1 [file medi-105-e50766-s001.pdf]

Supplementary Figure 1

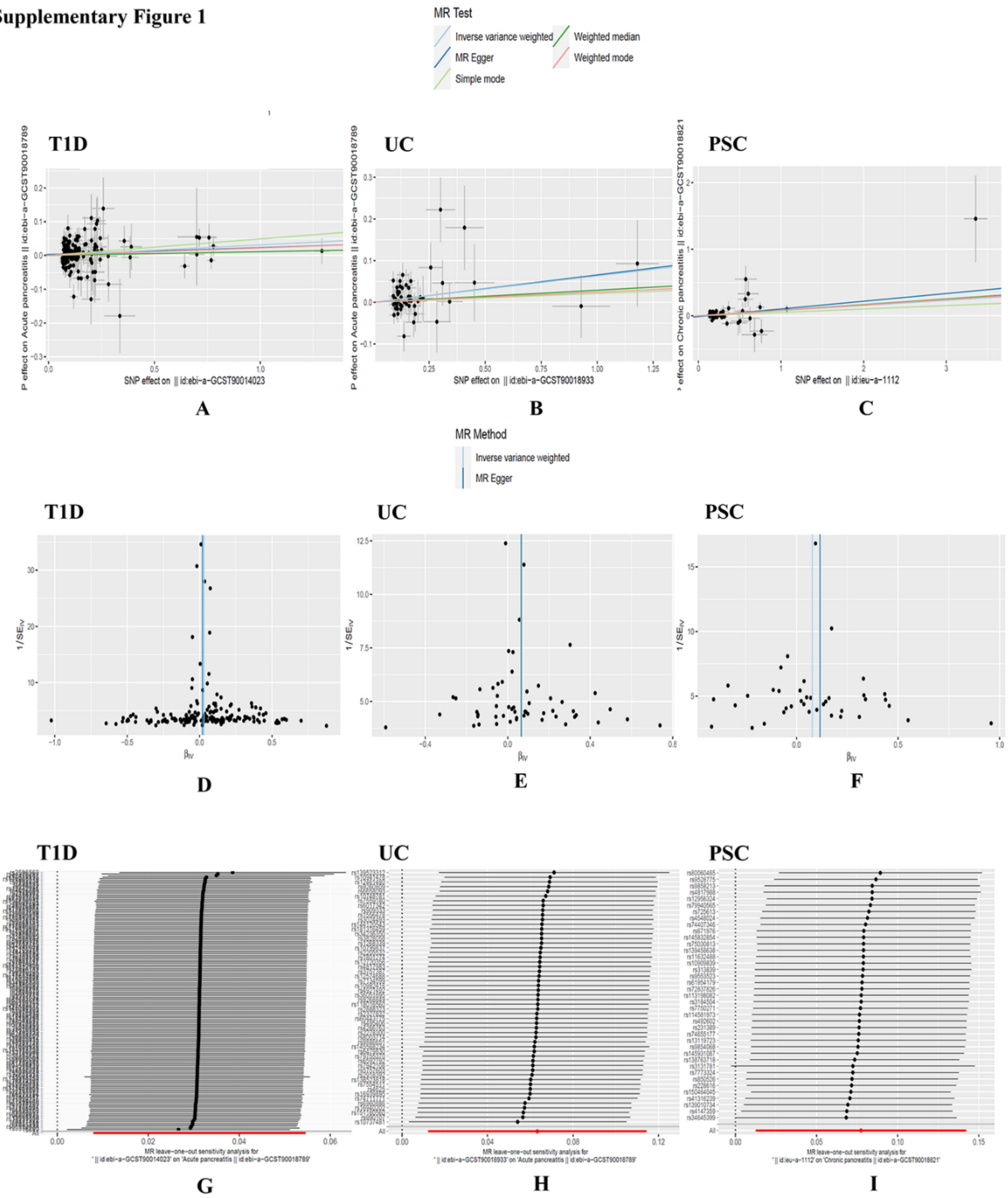

Supplementary Figure 1

(A-C). Scatter plots show the MR effect of each exposure on pancreatitis in different MR methods: (A) Causal estimates for T1D on AP; (B) Causal estimates for UC on AP; (C) Causal estimates for PSC on CP;

(D-F). Funnel plots for the causal relationship between autoimmune diseases and pancreatitis: (D) Causal estimates for T1D on AP; (E) Causal estimates for UC on AP; (F) Causal estimates for PSC on CP;

(G-I). Leave-one-out plots for the causal relationship between autoimmune diseases and pancreatitis: (G) Causal estimates for T1D on AP; (H) Causal estimates for UC on AP; (I) Causal estimates for PSC on CP.
