# Supplementary material for: Suggestive causal associations between 17 autoimmune diseases and pancreatitis: A two-sample Mendelian randomization study
Source: Medicine (Baltimore). 2026 Sep 18;105(38):e50766. doi: 10.1097/MD.0000000000050766 (PMC13593168; doi:10.1097/MD.0000000000050766)
Supplement: Supplementary file 2 [file medi-105-e50766-s002.pdf]

Supplementary Figure 2

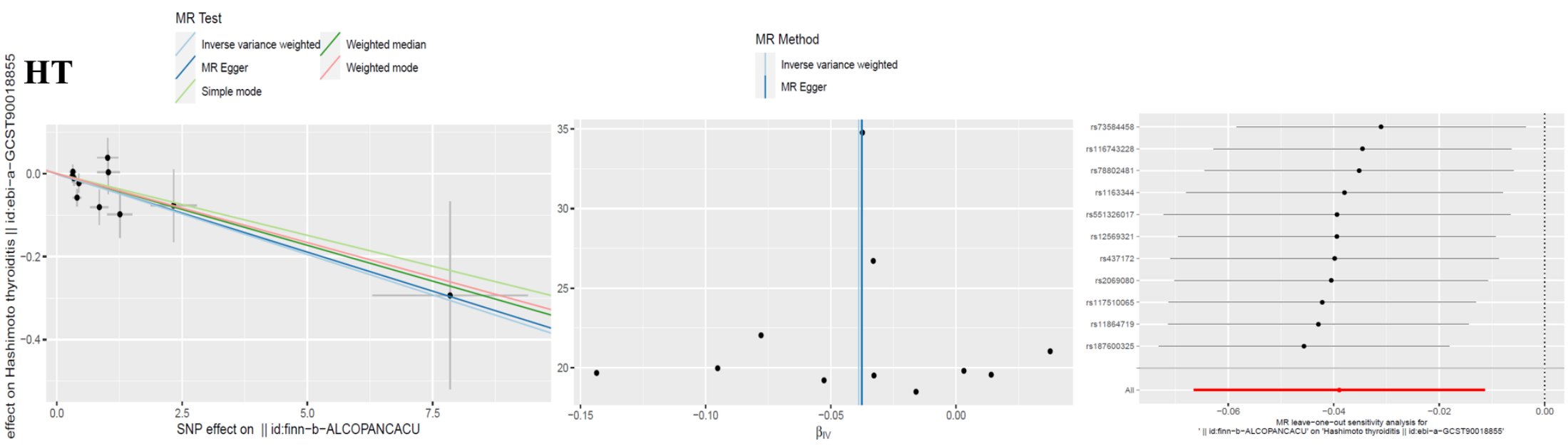

A **B** **C**

Supplementary Figure 2

A. Scatter plots show the MR effect of AAP on HT in different MR methods, Causal estimates for AAP on HT;

B. Funnel plots for the causal relationship between AAP and HT;

C. Leave-one-out plots for the causal relationship between AAP and HT.
