# Supplementary material for: Suggestive causal associations between 17 autoimmune diseases and pancreatitis: A two-sample Mendelian randomization study
Source: Medicine (Baltimore). 2026 Sep 18;105(38):e50766. doi: 10.1097/MD.0000000000050766 (PMC13593168; doi:10.1097/MD.0000000000050766)
Supplement: Supplementary file 3 [file medi-105-e50766-s003.pdf]

Supplementary table 1. Causal effects of 17 Autoimmune Diseases on AP

Exposure	SNP	R2	F	Inverse VarianceWeighted				Weighted Median				MR-Egger				P	P
				OR	(95%Cl)		P	OR	(95%Cl)		P	OR	(95%Cl)		P	pleiotropy	heterogeneity
					low	up			low	up			low	up			
Rheumatoid arthritis	148	0.007	68.28	1.041	0.966	1.123	0.290	1.084	0.963	1.219	0.181	1.211	1.000	1.466	0.052	0.608	0.178
Systemic lupuserythematosus	65	0.007	68.28	1.002	0.950	1.057	0.946	1.002	0.921	1.089	0.971	0.985	0.904	1.072	0.724	0.608	0.615
Eczema	260	0.000	50.31	1.064	0.673	1.682	0.790	1.164	0.575	2.357	0.673	1.898	0.645	5.584	0.246	0.247	0.268
Psoriasis	78	NA	78.7	1.014	0.978	1.052	0.449	1.028	0.964	1.095	0.404	1.000	0.934	1.070	0.993	0.616	0.435
Vitiligo	4	0.183	22.5	0.971	0.937	1.006	0.102	0.979	0.937	1.023	0.342	1.064	0.721	1.569	0.785	0.689	0.559
Multiple sclerosis	53	0.018	53.46	1.014	0.980	1.048	0.425	1.036	0.986	1.089	0.161	1.079	1.015	1.148	0.019	0.022	0.658
Myasthenia gravis	14	0.116	22.16	1.010	0.983	1.036	0.474	1.024	0.986	1.063	0.225	1.035	0.982	1.091	0.223	0.306	0.437
Narcolepsy	5	NA	33.71	0.917	0.830	1.013	0.087	0.935	0.822	1.063	0.306	0.977	0.565	1.689	0.939	0.831	0.638
Type 1 diabetes*	162	0.011	92.51	1.032	1.008	1.056	0.009	1.011	0.974	1.050	0.556	1.021	0.988	1.054	0.217	0.355	0.334
Grave disease	53	0.014	47.12	1.002	0.962	1.044	0.912	1.027	0.966	1.092	0.399	1.083	0.991	1.183	0.085	0.062	0.184
Hashimoto thyroiditis	48	0.003	50.5	1.036	0.982	1.092	0.199	1.048	0.969	1.133	0.244	1.033	0.918	1.162	0.596	0.955	0.069
Primary sclerosing cholangitis	39	0.034	68.64	1.036	0.996	1.078	0.077	1.055	0.997	1.117	0.063	1.007	0.937	1.082	0.852	0.357	0.109
Primary biliary cirrhosis	39	NA	53.63	0.999	0.971	1.028	0.941	0.981	0.942	1.022	0.358	0.958	0.907	1.011	0.128	0.084	0.650
Ulcerative colitis*	54	0.012	33.32	1.065	1.012	1.121	0.015	1.029	0.957	1.108	0.437	1.068	0.957	1.193	0.244	0.954	0.329
Crohn's disease	25	0.000	26.6	30.345	0.012	73788	0.391	1.936	0.000	170088	0.909	40.600	0.002	899395	0.475	0.926	0.373
Celiac disease	23	0.039	159.25	0.995	0.964	1.027	0.768	0.993	0.948	1.040	0.758	0.997	0.954	1.043	0.910	0.889	0.661
Autoimmune hepatitis	13	0.042	25.72	1.036	0.986	1.089	0.162	1.024	0.957	1.096	0.485	1.016	0.901	1.147	0.798	0.735	0.294

SNPs: single nucleotide polymorphisms; IVW: inverse variance weighted; WM: weighted median; MR-PRESSO: MR-pleiotropy residual sum and outlier; OR: odds ratio; CI: confidence interval
