# Supplementary material for: Suggestive causal associations between 17 autoimmune diseases and pancreatitis: A two-sample Mendelian randomization study
Source: Medicine (Baltimore). 2026 Sep 18;105(38):e50766. doi: 10.1097/MD.0000000000050766 (PMC13593168; doi:10.1097/MD.0000000000050766)
Supplement: Supplementary file 4 [file medi-105-e50766-s004.pdf]

Supplementary table 2. Causal effects of 17 Autoimmune Diseases on CP

Exposure	SNP	R2	F	Inverse VarianceWeighted				Weighted Median				MR-Egger				P pleiotropy	P heterogeneity
				OR	(95%Cl) low	(95%Cl) up	P	OR	(95%Cl) low	(95%Cl) up	P	OR	(95%Cl) low	(95%Cl) up	P		
**Rheumatoid arthritis**	148	0.007	68.28	1.094	0.972	1.232	0.137	1.110	0.925	1.331	0.262	1.048	0.772	1.421	0.765	0.140	0.750
**Systemic lupus erythematosus**	65	0.007	68.28	0.960	0.887	1.039	0.315	0.964	0.848	1.096	0.574	0.891	0.786	1.011	0.078	0.140	0.437
**Eczema**	260	0.000	50.31	0.971	0.478	1.974	0.935	1.960	0.635	6.049	0.242	2.268	0.416	12.361	0.345	0.281	0.524
**Psoriasis**	78	NA	78.7	1.048	0.986	1.114	0.134	1.017	0.917	1.129	0.743	0.995	0.889	1.113	0.927	0.284	0.225
**Vitiligo**	4	0.183	22.5	0.956	0.904	1.011	0.115	0.971	0.908	1.039	0.390	0.639	0.341	1.199	0.298	0.335	0.437
**Multiple sclerosis**	53	0.018	53.46	1.033	0.979	1.090	0.231	1.042	0.949	1.145	0.389	1.044	0.943	1.156	0.407	0.810	0.414
**Myasthenia gravis**	14	0.116	22.16	1.015	0.973	1.059	0.497	0.990	0.933	1.051	0.743	1.046	0.957	1.143	0.344	0.464	0.448
**Narcolepsy**	5	NA	33.71	0.986	0.842	1.153	0.856	0.950	0.787	1.146	0.592	1.160	0.492	2.735	0.757	0.730	0.721
**Type 1 diabetes**	161	0.011	92.51	0.987	0.948	1.027	0.505	0.961	0.902	1.025	0.230	0.980	0.927	1.036	0.472	0.729	0.045
**Grave disease**	53	0.014	47.12	1.029	0.952	1.112	0.474	1.063	0.962	1.174	0.231	1.109	0.929	1.325	0.257	0.358	0.001
**Hashimoto thyroiditis**	151	0.003	50.5	0.977	0.899	1.061	0.576	0.986	0.870	1.117	0.824	1.060	0.883	1.274	0.531	0.324	0.126
**Primary sclerosing cholangitis***	39	0.034	68.64	1.080	1.013	1.153	0.019	1.088	0.991	1.195	0.078	1.122	0.994	1.267	0.069	0.468	0.088
**Primary biliary cirrhosis**	39	NA	53.63	0.998	0.956	1.042	0.914	0.983	0.920	1.051	0.623	0.945	0.872	1.024	0.175	0.084	0.650
**Ulcerative colitis**	55	0.012	33.32	1.056	0.978	1.140	0.165	1.059	0.942	1.190	0.338	1.049	0.892	1.234	0.568	0.926	0.670
**Crohn's disease**	25	0.000	26.6	1.249	0.000	47469	0.967	90.964	0.000	118850176	0.530	1079	0.002	563233980	0.309	0.106	0.877
**Celiac disease**	23	0.039	159.25	0.995	0.940	1.053	0.861	1.038	0.969	1.111	0.290	1.038	0.961	1.121	0.354	0.136	0.010
**Autoimmune hepatitis**	13	0.042	25.72	0.997	0.891	1.115	0.953	0.996	0.882	1.124	0.944	1.077	0.816	1.422	0.612	0.560	0.010

SNPs: single nucleotide polymorphisms; IVW: inverse variance weighted; WM: weighted median; MR-PRESSO: MR-pleiotropy residual sum and outlier; OR: odds ratio; CI: confidence interval
