# Supplementary material for: Suggestive causal associations between 17 autoimmune diseases and pancreatitis: A two-sample Mendelian randomization study
Source: Medicine (Baltimore). 2026 Sep 18;105(38):e50766. doi: 10.1097/MD.0000000000050766 (PMC13593168; doi:10.1097/MD.0000000000050766)
Supplement: Supplementary file 5 [file medi-105-e50766-s005.pdf]

Supplementary table 3. Causal effects of 17 Autoimmune Diseases on AAP

Exposure	SNP	R2	F	Inverse VarianceWeighted				Weighted Median				MR-Egger				P	P
				OR	(95%Cl)		P	OR	(95%Cl)		P	OR	(95%Cl)		P	pleiotropy	heterogeneity
					low	up			low	up			low	up			
**Rheumatoid arthritis**	146	0.007	68.28	0.975	0.763	1.247	0.842883171	0.969	0.678	1.385	0.862	1.208	0.646	2.260	0.554	0.357	0.804
**Systemic lupus erythematosus**	60	0.007	68.28	0.955	0.800	1.140	0.61035247	0.899	0.667	1.212	0.486	0.853	0.634	1.148	0.299	0.357	0.206
**Eczema**	253	0.000	50.31	0.832	0.207	3.349	0.795673438	0.603	0.070	5.153	0.644	0.141	0.005	4.005	0.253	0.254	0.797
**Psoriasis**	73	NA	78.7	1.020	0.910	1.143	0.735106936	1.028	0.854	1.237	0.774	0.965	0.785	1.186	0.733	0.529	0.505
**Vitiligo**	4	0.183	22.5	0.970	0.870	1.082	0.587840769	0.965	0.849	1.097	0.590	0.628	0.194	2.035	0.519	0.542	0.840
**Multiple sclerosis**	51	0.018	53.46	0.956	0.856	1.066	0.415211997	0.948	0.805	1.116	0.523	1.003	0.821	1.225	0.977	0.573	0.180
**Myasthenia gravis**	12	0.116	22.16	0.985	0.906	1.071	0.717111059	1.042	0.926	1.174	0.493	1.059	0.894	1.254	0.525	0.358	0.454
**Narcolepsy**	5	NA	33.71	1.321	0.923	1.891	0.12825945	1.219	0.835	1.779	0.305	0.687	0.080	5.921	0.755	0.588	0.223
**Type 1 diabetes**	159	0.011	92.51	0.969	0.903	1.040	0.382862443	1.017	0.910	1.136	0.768	0.977	0.886	1.078	0.648	0.808	0.789
**Grave disease**	53	0.014	47.12	0.954	0.839	1.085	0.475581575	0.903	0.755	1.079	0.261	1.026	0.765	1.375	0.867	0.595	0.137
**Hashimoto thyroiditis**	145	0.003	50.5	1.066	0.913	1.245	0.41738581	1.005	0.767	1.316	0.973	1.043	0.748	1.454	0.804	0.885	0.619
**Primary sclerosing cholangitis**	39	0.034	68.64	1.033	0.912	1.171	0.606491756	1.036	0.873	1.229	0.685	0.932	0.742	1.170	0.546	0.293	0.089
**Primary biliary cirrhosis**	39	NA	53.63	1.091	0.999	1.191	0.05354954	1.097	0.958	1.256	0.182	0.966	0.817	1.142	0.686	0.103	0.520
**Ulcerative colitis**	54	0.012	33.32	1.152	0.980	1.356	0.086732462	1.151	0.918	1.442	0.223	1.007	0.612	1.657	0.977	0.577	0.713
**Crohn's disease**	25	0.000	26.6	5.699	0.000	391630419	0.850075581	0.052	0.000	115653008	0.788	5.015	0.000	25737290917	0.889	0.985	0.651
**Celiac disease**	23	0.039	159.25	0.950	0.855	1.055	0.333780041	0.947	0.820	1.092	0.452	0.891	0.770	1.031	0.135	0.231	0.608
**Autoimmune hepatitis**	13	0.042	25.72	1.031	0.880	1.207	0.706932017	0.959	0.789	1.164	0.670	1.360	0.974	1.898	0.098	0.098	0.155

SNPs: single nucleotide polymorphisms; IVW: inverse variance weighted; WM: weighted median; MR-PRESSO: MR-pleiotropy residual sum and outlier; OR: odds ratio; CI: confidence interval
