# Supplementary material for: Suggestive causal associations between 17 autoimmune diseases and pancreatitis: A two-sample Mendelian randomization study
Source: Medicine (Baltimore). 2026 Sep 18;105(38):e50766. doi: 10.1097/MD.0000000000050766 (PMC13593168; doi:10.1097/MD.0000000000050766)
Supplement: Supplementary file 6 [file medi-105-e50766-s006.pdf]

Supplementary table 4. Causal effects of 17 Autoimmune Diseases on ACP

Exposure	SNP	R2	F	Inverse VarianceWeighted				Weighted Median				MR-Egger				P	P
				OR	(95%Cl)		P	OR	(95%Cl)		P	OR	(95%Cl)		P	pleiotropy	heterogeneity
					low	up			low	up			low	up			
**Rheumatoid arthritis**	146	0.007	68.28	1.085	0.924	1.274	0.317164849	1.018	0.801	1.292	0.886	0.885	0.588	1.331	0.558	0.359	0.505
**Systemic lupus erythematosus**	60	0.007	68.28	0.986	0.872	1.114	0.817904772	0.937	0.766	1.145	0.525	0.912	0.743	1.120	0.383	0.359	0.410
**Eczema**	253	0.000	50.31	1.035	0.395	2.712	0.943739	0.838	0.190	3.692	0.815	1.006	0.099	10.181	0.996	0.979	0.494
**Psoriasis**	73	NA	78.7	1.047	0.967	1.134	0.256297274	1.081	0.954	1.225	0.222	1.076	0.931	1.243	0.327	0.664	0.426
**Vitiligo**	4	0.183	22.5	1.031	0.956	1.112	0.42385905	1.025	0.942	1.116	0.569	1.259	0.558	2.837	0.635	0.677	0.858
**Multiple sclerosis**	51	0.018	53.46	0.948	0.878	1.024	0.177741487	1.004	0.902	1.118	0.936	1.018	0.885	1.170	0.805	0.239	0.122
**Myasthenia gravis**	12	0.116	22.16	1.005	0.937	1.076	0.898601929	0.995	0.916	1.081	0.904	0.974	0.843	1.126	0.731	0.643	0.154
**Narcolepsy**	5	NA	33.71	1.079	0.874	1.332	0.479130024	0.979	0.746	1.285	0.878	1.145	0.300	4.379	0.855	0.935	0.136
**Type 1 diabetes**	159	0.011	92.51	0.954	0.909	1.002	0.060091188	0.938	0.864	1.018	0.127	0.941	0.879	1.007	0.081	0.561	0.635
**Grave disease**	53	0.014	47.12	1.014	0.921	1.116	0.780952694	1.002	0.887	1.132	0.971	1.136	0.914	1.412	0.257	0.259	0.023
**Hashimoto thyroiditis**	145	0.003	50.5	1.063	0.948	1.192	0.296112558	1.079	0.901	1.292	0.406	1.119	0.875	1.432	0.371	0.642	0.116
**Primary sclerosing cholangitis**	39	0.034	68.64	0.968	0.895	1.047	0.414082451	0.901	0.800	1.015	0.087	0.972	0.841	1.123	0.701	0.947	0.330
**Primary biliary cirrhosis**	39	NA	53.63	1.057	0.988	1.131	0.105615964	1.000	0.906	1.104	0.999	0.930	0.825	1.049	0.247	0.019	0.163
**Ulcerative colitis**	54	0.012	33.32	1.020	0.899	1.158	0.753656913	0.921	0.781	1.087	0.332	0.918	0.621	1.358	0.671	0.789	0.090
**Crohn's disease**	25	0.000	26.6	32.643	0.000	23983079	0.613003476	1.752	0.000	4191929	0.940	8.110	0.000	211441215	0.812	0.218	0.248
**Celiac disease**	23	0.039	159.25	0.937	0.872	1.007	0.077485406	0.964	0.878	1.058	0.440	0.981	0.887	1.085	0.707	0.218	0.475
**Autoimmune hepatitis**	13	0.042	25.72	1.018	0.911	1.137	0.758527443	0.971	0.858	1.100	0.647	1.272	1.016	1.593	0.060	0.054	0.136

SNPs: single nucleotide polymorphisms; IVW: inverse variance weighted; WM: weighted median; MR-PRESSO: MR-pleiotropy residual sum and outlier; OR: odds ratio; CI: confidence interval
