# Supplementary material for: Suggestive causal associations between 17 autoimmune diseases and pancreatitis: A two-sample Mendelian randomization study
Source: Medicine (Baltimore). 2026 Sep 18;105(38):e50766. doi: 10.1097/MD.0000000000050766 (PMC13593168; doi:10.1097/MD.0000000000050766)
Supplement: Supplementary file 7 [file medi-105-e50766-s007.pdf]

Supplementary table 5. Reverse MR analysis： Causal effects of pancreatitis on 17 Autoimmune Diseases

Exposure	Inverse VarianceWeighted（AP ）				Inverse VarianceWeighted（CP）				Inverse VarianceWeighted（AAP）				Inverse VarianceWeighted（ACP）			
	OR	(95%Cl)		P	OR	(95%Cl)		P	OR	(95%Cl)		P	OR	(95%Cl)		P
		low	up			low	up			low	up			low	up	
**Rheumatoid arthritis**	1.085	0.924	1.274	0.317	1.085	0.924	1.274	0.317165	1.085	0.924	1.274	0.317	1.085	0.924	1.274	0.317
**Systemic lupus erythematosus**	0.986	0.872	1.114	0.818	0.986	0.872	1.114	0.817905	0.986	0.872	1.114	0.818	0.986	0.872	1.114	0.818
**Eczema**	1.035	0.395	2.712	0.944	1.035	0.395	2.712	0.943739	1.035	0.395	2.712	0.944	1.035	0.395	2.712	0.944
**Psoriasis**	1.047	0.967	1.134	0.256	1.047	0.967	1.134	0.256297	1.047	0.967	1.134	0.256	1.047	0.967	1.134	0.256
**Vitiligo**	1.031	0.956	1.112	0.424	1.031	0.956	1.112	0.423859	1.031	0.956	1.112	0.424	1.031	0.956	1.112	0.424
**Multiple sclerosis**	0.948	0.878	1.024	0.178	0.948	0.878	1.024	0.177741	0.948	0.878	1.024	0.178	0.948	0.878	1.024	0.178
**Myasthenia gravis**	1.005	0.937	1.076	0.899	1.005	0.937	1.076	0.898602	1.005	0.937	1.076	0.899	1.005	0.937	1.076	0.899
**Narcolepsy**	1.079	0.874	1.332	0.479	1.079	0.874	1.332	0.47913	1.079	0.874	1.332	0.479	1.079	0.874	1.332	0.479
**Type 1 diabetes**	0.954	0.909	1.002	0.060	0.954	0.909	1.002	0.060091	0.954	0.909	1.002	0.060	0.954	0.909	1.002	0.060
**Grave disease**	1.014	0.921	1.116	0.781	1.014	0.921	1.116	0.780953	1.014	0.921	1.116	0.781	1.014	0.921	1.116	0.781
**Hashimoto thyroiditis**	1.063	0.948	1.192	0.296	1.063	0.948	1.192	0.296113	1.063	0.948	1.192	0.296	1.063	0.948	1.192	0.296
**Primary sclerosing cholangitis**	0.968	0.895	1.047	0.414	0.968	0.895	1.047	0.414082	0.968	0.895	1.047	0.414	0.968	0.895	1.047	0.414
**Primary biliary cirrhosis**	1.057	0.988	1.131	0.106	1.057	0.988	1.131	0.105616	1.057	0.988	1.131	0.106	1.057	0.988	1.131	0.106
**Ulcerative colitis**	1.020	0.899	1.158	0.754	1.020	0.899	1.158	0.753657	1.020	0.899	1.158	0.754	1.020	0.899	1.158	0.754
**Crohn's disease**	32.643	0.000	23983079.649	0.613	32.643	0.000	23983079.649	0.613003	32.643	0.000	23983079.649	0.613	32.643	0.000	23983079.649	0.613
**Celiac disease**	0.937	0.872	1.007	0.077	0.937	0.872	1.007	0.077485	0.937	0.872	1.007	0.077	0.937	0.872	1.007	0.077
**Autoimmune hepatitis**	1.018	0.911	1.137	0.759	1.018	0.911	1.137	0.758527	1.018	0.911	1.137	0.759	1.018	0.911	1.137	0.759

SNPs: single nucleotide polymorphisms; IVW: inverse variance weighted; WM: weighted median; MR-PRESSO: MR-pleiotropy residual sum and outlier; OR: odds ratio; CI: confidence interval
